# Effect of Lipids and Lipoproteins on Hematopoietic Cell Metabolism and Commitment in Atherosclerosis

**DOI:** 10.20900/immunometab20210014

**Published:** 2021-03-29

**Authors:** Andrea Baragetti, Fabrizia Bonacina, Alberico Luigi Catapano, Giuseppe Danilo Norata

**Affiliations:** 1 Department of Excellence of Pharmacological and Biomolecular Sciences, Università degli Studi di Milano, Milan 20133, Italy; 2 IRCCS Multimedica, Milan 20138, Italy; 3 Centro SISA per lo Studio dell’Aterosclerosi, Ospedale Bassini, Cinisello Balsamo 20092, Italy

**Keywords:** hematopoiesis, atherosclerosis, cholesterol, cellular metabolism

## Abstract

Hematopoiesis is the process that leads to multiple leukocyte lineage generation within the bone marrow. This process is maintained throughout life thanks to a nonstochastic division of hematopoietic stem cells (HSCs), where during each division, one daughter cell retains pluripotency while the other differentiates into a restricted multipotent progenitor (MPP) that converts into mature, committed circulating cell. This process is tightly regulated at the level of cellular metabolism and the shift from anaerobic glycolysis, typical of quiescent HSC, to oxidative metabolism fosters HSCs proliferation and commitment. Systemic and local factors influencing metabolism alter HSCs balance under pathological conditions, with chronic metabolic and inflammatory diseases driving HSCs commitment toward activated blood immune cell subsets. This is the case of atherosclerosis, where impaired systemic lipid metabolism affects HSCs epigenetics that reflects into increased differentiation toward activated circulating subsets.

Aim of this review is to discuss the impact of lipids and lipoproteins on HSCs pathophysiology, with a focus on the molecular mechanisms influencing cellular metabolism. A better understanding of these aspects will shed light on innovative strategies to target atherosclerosis-associated inflammation.

## Introduction

Atherosclerosis results from the deposit of cholesterol within the intima of the arterial wall, an event that promotes inflammation and immune cell recruitment in the vessels, thus promoting atherosclerotic plaque formation. Monocytes-derived macrophages and activated T cells are the most abundant infiltrated immune cells in human atherosclerotic plaques, especially in vulnerable and unstable plaques. Parallel to local immune activation, systemic changes in the number, proportion and function of immune cells have been reported in patients with both stable and acute atherosclerotic cardiovascular disease (ASCVD), thus strengthening the association between the disease and a systemic immuno-inflammatory response [1–6]. Besides, the results from recent clinical trials with anti-inflammatory therapies (CANTOS [7], COLCOT [8] and LoDoCo2 [9]) have contributed to demonstrate the causality of this association, suggesting that the activation of immune response is not merely a bystander of lipid overload, but instead is actively involved in disease progression. Furthermore, emerging evidences show that the pro-inflammatory activation of immune cells occurs already at the level of hematopoietic precursors even in the bone marrow by mechanisms of functional priming (the so called “trained immunity”) and/or clonal hematopoiesis, that were shown to increase the risk of atherosclerotic cardiovascular disease [10,11]. This arises as the consequence of the connection between classical risk factors such as hypercholesterolemia or hyperglycemia and alterations in bone marrow cells characteristics/epigenetics which translate into sustained hematopoiesis and increased commitment toward activated immune subsets. This review aims at discussing the current knowledge on the metabolic adaptations occurring in hematopoietic cells during atherosclerosis and how this influences myeloid compartment homeostasis. A deeper understanding of these mechanisms will set the stage for testing possibilities to target cellular metabolism to reprogram both hematopoietic cells as well as immune cells as a strategy to improve atherosclerosis-associated inflammation.

## Physiological Regulation of Hematopoiesis

**Figure 1:**
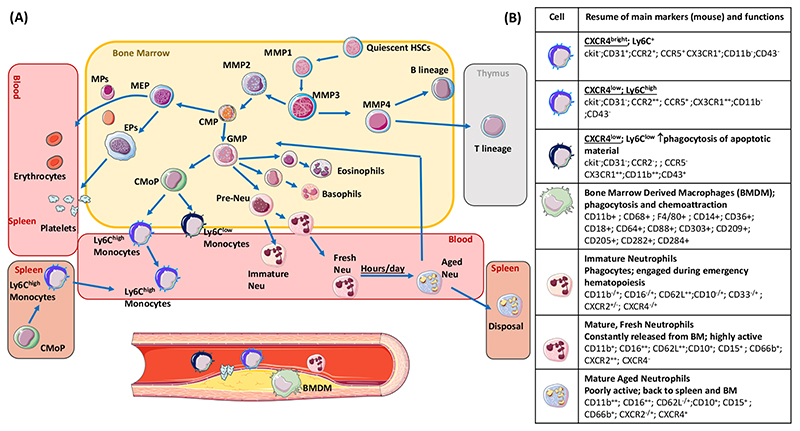
Hematopoiesis. (**A**) Hematopoietic, precursor cells and different cellular lineages in the bone marrow and in extra-medullary tissues (thymus and spleen) (cells and markers for mice are reported). “HSCs”-hematopoietic stem cell; “MPP”, Multipotent Progenitor Cell; “CMP”, Common Myeloid Progenitor; “GMP”, Granulocyte/Macrophage Progenitors; “CMoP”, Common monocyte progenitor; “MEP”, Megakaryocyte–Erythroid Progenitor; “EP”, Erythroid Progenitor; “MEP”, Megakaryocyte Progenitor; “Neu”, Neutrophil. The direction of light-blue arrows indicates the progression/proliferation towards the downstream immune cell. (**B**) Murine markers and key functions (in bold) for each immune cell subset. Markers are identified as: “neg” (negative, cell not expressing the marker); “low” (cell poorly expressing the marker); “+” positive (cell expressing the marker); “bright” cell highly-expressing the specific marker.

Hematopoiesis is the physiological process by which a small pool of Hematopoietic Stem Cells (HSCs), characterized by self-renewal and pluripotency, produces the variety of red blood cells and immune-competent leukocytes circulating within our blood. Hematopoiesis is maintained lifelong thanks to the asymmetric scheme of division rate, where one daughter cell remains an HSC and the other differentiates into a restricted progenitor with limited self-renewal capacity. From these progenitor cells, downstream precursors differentiate and proliferate in order to provide different lineages in a nonstochastic hierarchy (Figure 1).

In humans, HSC are around 3000–10,000 per femur and their division rate is estimated between once every three months to once every year [12,13]. Therefore, a proportion of HSCs maintain a relatively constant phenotype of quiescence, while a small fraction differentiates towards multipotent progenitors (MPPs), which are characterized by massively increased differentiating potential and which will produce a nonstochastic proportion of downstream cellular products [14,15]. Single-cell RNA techniques recently highlighted that multiple MPPs exist which, despite all originating from a firstly committed MPP1 [16], develop an a priori engagement towards multiple lineage outputs (e.g., MPP2 specialize for development of the myeloid, erythroid and megakaryocyte lineages, while MPP3 evolve to lymphoid progenitors). This guarantees 4500 to 11,000 circulating leukocytes per cubic millimeter (55–70% as neutrophils [17]) in physiological conditions and a massive increase in the number of circulating neutrophils and monocytes under inflamed conditions, including myocardial infarction (MI), in mice models [18,19] and humans [20]. This dynamic adaptation of the hematopoietic system provides an enormous amount of heterogeneous leukocyte subfractions and is driven by a storm of neurochemical mediators (β3 adrenergic system activation), cytokines (IL-1beta, IL-6, TNF-alpha, damage-associated molecular patterns, calprotectin S100A8/A9) and bone marrow mobilizers (Granulocytes-Monocytes Colony Stimulating Factor, GM-CSF; and Stromal Cell-Derived Factor 1, SDF-1).

Beyond the acute response of the bone marrow compartment, it is emerging how HSCs long-term lifespan, their proliferative potential and the differentiation to downstream progenitors is affected also by long term exposure to cardio-metabolic risk factors, including hypercholesterolemia, hyperglycemia as well as daily and recurrent exposure to hypercaloric foods; this latter indeed, by promoting excessive increase of glucose and lipid levels after food intake, the so called “postprandial hyperlipemia”, is considered a cardio-metabolic and inflammatory condition independently associated with elevated cardiovascular risk [21–26]).

## Environmental Factors Influencing Hematopoietic Cell Homeostasis

**Figure 2:**
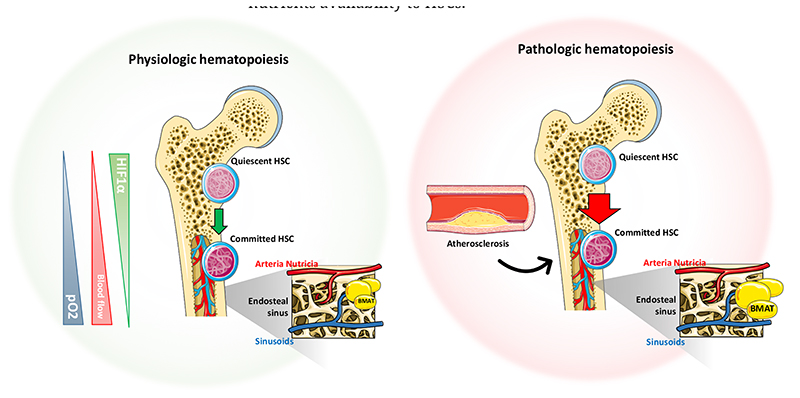
Bone marrow vascular network and factors affecting hematopoiesis. Anatomical description of the principal medullary factors influencing the proliferation and commitment of quiescent HSC (“Hematopoietic Stem Cell”) in physiological (**left**) or in pathological conditions (**right**). Triangles on the left side of the picture indicate the relative abundance of factors affecting the quiescent to committed HSC transition. The magnified inset on the right side of each femur highlights the vascular interaction between the arterial and venous network at the level of the endosteal sinus under physiological and pathological conditions.

Within the bone marrow, HSCs reside in close contact to the vascular network. The majority of non-dividing quiescent HSCs are located in close proximity to the sinusoids and adhere to the endosteum (an area where oxygen tension is minimal, around 55 mmHg pO_2_ [27]), while 10–20% of HSCs are located near the arterioles (where the oxygen tension is equal to that of the bloodstream) [28–30]. Anatomically, the central artery (“arteria nutricia”) penetrates the bone marrow through the nutrient canal and divides into an ascending and descending branch; within the endosteal space, it extends as thin-walled arterioles and follows the long axis over the metaphysis where ends with a complex ramification at the epiphysis, including the medullary sinusoids, an important reticular network of fenestrated vessels over diaphysis and metaphysis (Figure 2). This structure connects the arterial structure to the venous sinusoids, which drain the cellular and molecular material into the central venous system towards the nutrient vein. As such the vascular network is fundamental in mediating the influx and efflux of the hematopoietic and non-hematopoietic cells from the circulation but also controls oxygen and nutrients availability to HSCs.

HSCs, located in areas with a low oxygen tension (hypoxic regions), rely on the activation of hypoxia inducible Factor 1 alpha (HIF1-alpha) [31,32], the master transcriptional regulator of different genes encoding for glycolytic enzymes (LDHA, PKM2, GLUT1, PFKL and PDK2) [33], this, coupled to the availability of glucose, is crucial to support quiescent HSCs energy demand via anaerobic glycolysis. HIF1-alpha senses the reduced pO_2_ and, through the dimerization of the alpha and beta subunits, it favors the activity of the pyruvate dehydrogenase kinases (PDK) and, in turn, inactivates the pyruvate dehydrogenase. This is a key mechanism which limits TCA cycle flux and oxidative phosphorylation (OXPHOS). This activity of HIF1-alpha decreases in HSCs located in more oxygenated medullary areas and this promotes the cellular metabolic shifts from anaerobic glycolysis to mitochondrial oxidative metabolism and supports the differentiation towards downstream effectors [34].

Limiting oxidative metabolism minimizes the possibility to generate radical oxygen species (ROS) and DNA damage, this aspect becomes relevant over lifespan, as HSCs becomes less efficient to scan for and repair damages of the genomic heritage. This appears to be relevant when somatic mutations occur in specific loci such as the epigenetic regulators TET2 (ten-eleven translocation 2), DNMT3 (DNA nucleotide methyltransferase 3A), or ASXL1 (the addition of sex combs like 1) as they favor the selection and expansion of a pool of HSCs with a competitive advantage during hematopoiesis over the rest of the other stem/progenitor cells [35]. This process, which is called clonal hematopoiesis of indeterminate potential (CHIP), has been significantly associated with elevated atherosclerotic burden in experimental models of hypercholesterolemia and atherosclerosis [10,36]. In this experimental setting, TET2 deficiency profoundly changed the transcriptome of myeloid cells and bone marrow derived macrophages, which displayed an enrichment in RNA classes coding for cytokines/chemokines receptors, coupled to the reduction of genes involved in lysosomal function. Atheroprone mice receiving TET2 deficient bone marrow under high fat/high cholesterol diet, presented atherosclerotic plaque enriched in pro-inflammatory macrophages [37]. Of note, a cluster of variants in CHIP-associated loci were associated with increased risk of coronary artery disease in different cohorts [10].

Another key factor that is emerging as a critical contributor to HSC physiology is the amount of adipose tissue that accumulate in the bone marrow (BMAT) [38,39]. HSC number inversely correlates with the amount of adipocytes resident in the parenchymal structure of bones and, over lifespan, hematopoietic red bone marrow is replaced by fatty yellow marrow with HSCs being less efficient to differentiate into downstream precursors cells [40]. Recently it has been shown that lipid droplets budding from BMAT interact with phagocytes around sinusoids and support the maturation of erythroblasts, myeloid cells and, to a lesser extent, granulocytes [41].

In addition, also metabolites generated from the processing of highly-caloric nutrients by the microbiota, contribute to hematopoiesis. Different gut microbiota derived metabolites have been shown to impact HSCs commitment [42] and to determine circadian replenishment of the patrolling pool of phagocytes and neutrophils in peripheral tissues [43]. Selective gut microbiota species orchestrate myeloid and granulocytic hierarchal clustering by producing specific fatty acids [42] (e.g., butyrate [44]). This process is promoted by the production of phospholipids from multiple dietary sources or substrates which are oxidized in the liver and, in response to inflammatory conditions, foster myeloid commitment. This is the case for trimethylamine N-oxide (TMAO), which originates from the metabolism of intestinally absorbed choline and l-carnitine and has been associated to both atherogenesis [45,46] and to increased circulating myeloid cell levels[47].

**Figure 3:**
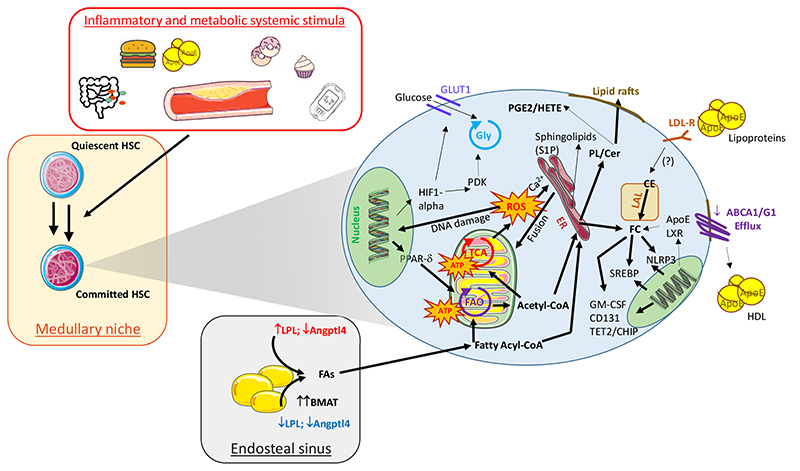
Impact of systemic and cellular lipid metabolism on HSCs commitment in atherosclerosis. Main cellular immune-metabolic circuits involved in HSC commitment during atherogenesis. Cellular pathways with enhanced expression/activity are indicated with bold arrows while those down-regulation or with reduced activity are represented with thin arrows. “Gly”, Glycolysis; “TCA”, (Tricarboxylic Acid Cycle); “FAO”, Fatty Acids Oxygenation; “ATP”, Adenosine Tri-Phosphate; “GLUT-1”, Glucose Transporte-1 isoform; “HIF1-alpha”, Hypoxia Inducible Factor 1 alpha; “PDK”, Pyruvate Dehydrogenase Kinase; “PPAR-δ”, Peroxisome proliferator-activated receptor delta; “ROS”, Reactive Oxygen Radical species; “ER”, Endoplasmic Reticulum; “PL” Phospholipids; “Cer”, Ceramids; “LAL”, Lysosomial Acid Lipase; “CE”, Cholesterol Esters; “LXR”, Liver-X-Receptors”; “NLRP3”, NOD-Like Receptor-3; “SREBP”, Sterol regulatory element-binding proteins; “GM-CSF”, granulocyte-macrophage colony-stimulating factor; “TET2”, ten-eleven translocation 2; “CHIP”, clonal haematopoiesis of indeterminate potential; “LDL-R”, Low-Density Lipoproteins Receptor; “ApoE”, Apolipoprotein E; “ABCG/A”, ATP-Binding Cassette transporter G/A isoforms; “LPL”, Lipoprotein Lipase; “FAs” Fatty Acids; “BMAT”, Bone Marrow Adipose Tissue; “Angptl4”, Angiopoietin Like 4 protein.

Therefore, changes in environmental conditions and nutrients supply affect hematopoietic niches homeostasis and thus modulate HSC plasticity including their inflow in the bloodstream as well as their functional commitment. These processes rely on the reprograming of cellular metabolism which shift from that of quiescent and poorly energy demanding HSC towards a more energy-demanding proliferative phenotype (Figure 3). Only recently we have started appreciating the cross talk between nutrients supply and cellular utilization, suggesting that a deeper knowledge in this field is crucial to identify key metabolic checkpoints that could be targeted to promote HSCs immune-metabolic reprogramming during atherosclerosis and cardiovascular diseases.

## Cellular Energetic Circuits Involved in Hematopoietic Cell Commitment in Inflammatory Conditions

HSC mobilization to more oxygenated areas during their differentiation is associated with cellular metabolism rewiring and increased OXPHOS activity. The preferential oxidation of FA (FAO) improves hematopoietic specification by acetyl-CoA-dependent histone modifications [48] and also increases mitochondrial remodelling that supports the asymmetric division of HSCs. The availability of FAs is related to the hydrolysis of glycerolipids and triacylglycerols (TG) stored in the BMAT and, at the molecular level, promotes the activation of the promyelocytic leukemia (PML)–peroxisome proliferator-activated receptor δ (PPAR-δ)–fatty-acid oxidation (FAO) axis [49]. Although the use of FAO more likely provides higher energetic yield (as the oxidation of one palmitate molecule generates 129 ATP molecules) [50], an excessive engagement of this process promotes ROS production, oxidative stress response [49] and the selective degradation of mitochondria (mitophagy) [51].

Elevated availability of saturated FAs in the HSCs dampens the activity of autophagy related 5 (Atg5), a key protein involved in the extension of the phagophoric membrane in autophagic vesicles, and this results into reduced cellular mitochondrial cellular mass of hematopoietic cells committed to become lymphocytes, in favor of a preferential expansion of the myeloid compartment. Also, Atg5 repression in myeloid lineage aggravates atherosclerosis and negatively impact on the inflammatory composition of atherosclerotic lesions in mice fed on diet enriched in polyunsaturated fats (PUFAs) [52], lipid dietary sources that favors the activity of macrophage autophagy [53]. Accordingly, myeloid skewing of hematopoietic cells appears proportional to the quantity of white adipose tissue volume in the mid-shaft of the bones [54,55]. Still whether BMAT, whose increased volume correlates with extent of aortic atherosclerotic calcification and predicts occurrence of atherosclerotic cardiovascular events independently from risk factors [56], acts as site of energy storage to support bone marrow function and maturation towards myeloid subsets, or negatively regulate HSCs mobilization is heavily investigated.

Additional mechanisms support hematopoietic differentiation under inflamed and atherosclerosis related conditions. Excessive glucose uptake support HSCs inflammatory skewing, and indeed glucose transporter (Glut1) deficiency in bone marrow cells limits excessive myelopoiesis and accelerated atherosclerosis in experimental models [57]. Besides to provide faster ATP replenishment, the switch to aerobic consumption of glucose coincides with the accumulation of TCA metabolites, that could restrain the hematopoietic pluripotency instead of being used as energetic substrates [58]. This effect seems to depend on the action of ATP citrate lyase (ACLY), that by converting mitochondrial citrate to Acetyl-CoA, seizes Acetyl-CoA from the TCA cycle diverting the molecule to histone acetylation and cholesterol synthesis [59]. Similar epigenetic mechanisms have been described for TCA’s derived alpha-ketoglutarate [60] further confirming the dependency between energetic setting and epigenetic profile. This control extends also to checkpoints of cellular metabolism, as the AMP-activated protein kinase (AMPK), that phosphorylates and stabilizes TET2. Vice versa, this ability is blunted under insulin resistance and diabetes, where AMPK activity is reduced [61], thus leading to clonal HSCs expansion and elevated risk of atherosclerotic diseases [13].

It is worth to speculate whether these epigenetic adaptations, such as histone modifications [62], could persist even in differentiated cells, thus affecting atherosclerosis progression by forcing prolonged differentiation over time into more aggressive immune cells [63]. This “epigenetic trained phenotype” has been confirmed in vivo, since bone marrow-derived myeloid cells collected from mice fed to high-cholesterol diet persistently maintain the augmented activated immune-inflammatory potential when transplanted in mice fed to standard diet. In line with this, the reported ACLY hyper-activation in human atherosclerotic plaque could be the result of hematopoietic imprinting and indeed its specific deficiency in the myeloid lineage (LysM positive cells) stabilizes atherosclerotic plaque progression while its activation increases the transcription of LPS-induced gene expression in cultured macrophages. In addition, also a “leaky-gut” enhances the absorption of inflammatory metabolites produced by the microbiota (e.g., LPS) which were shown to promote epigenetic adaptations on the myeloid lineage [64], to foster the proliferation of pro-inflammatory monocytes (in mice as Ly6Chigh and in humans as CD14^+^/C16^++^) and to induce atherothrombosis through TLR4-mediated neutrophilic cathepsin G activation [65]. Of note this phenotype is also associated to the activation of NLRP3, a component of the inflammasome that triggers inflammatory response by promoting IL-1β release [66] and fuels myeloid hyperactivity in atherosclerosis [67,68]. Indeed, TLR4 inhibition dampens NRPL3 priming and reverts the pro-inflammatory phenotype of neutrophils [65]. Similarly, inhibition of NRPL3 inflammasome by beta-glucan exposure of LPS-treated bone marrow derived macrophages derails their inflammatory phenotype induced by epigenetic modifications, including accumulation of active histone marks at promoter and enhancers of genes in the lipid metabolism and phagocytic pathways [64]. Together these evidences surmise the appealing possibility that the hyperactivation of the NLRP3 pathway, an evolutionary-conserved tool for the adaptation of the hematopoietic system and the myeloid compartment of the host organism facing environmental stimuli, might represent not only a target for therapeutic strategies (e.g., past experiences of the CANTOS trial), but also a tool for personalized cardiovascular risk stratification [68].

Therefore, cellular metabolic plasticity drives the fate of the hematopoietic tree, in response to the environmental settings and to the availability of energetic supplies. While oxidation of FA is physiologically required for hematopoietic specification in physiology, the oxidative metabolism consequent to either abundant lipids availability or increased glycolytic flux contribute to HSC commitment. The exacerbation of the latter process co-opts the multipotent downstream progenitors toward myeloid compartment, which will be more likely prompt to support the inflammatory response associated to atherosclerosis.

## Impact of Intracellular Lipid Synthesis and Extracellular Lipid Uptake On Hematopoietic Cell Commitment During Atherosclerosis

Lipids are crucial for both HSC self-renewal as well as HSC asymmetric differentiation and commitment not only as fuel but also as macromolecules for building new membranes (such as is the case for cholesterol and FA) [41]. Intriguingly, both lipid synthesis and lipid uptake pathways are activated in HSC and become overactivated during inflammatory conditions including atherosclerosis [69]. The reason why both processes are activated and whether they are redundant or not for HSCs commitment during atherosclerosis is still debated.

Key extracellular receptors responding to signals devoted to control HSCs proliferation and commitment are located in lipid rafts, cholesterol enriched area in the membrane. Alterations in the mechanisms controlling cholesterol availability in lipids rafts have been associated with a profound impact on HSCs physiology. The deficiency of apolipoprotein E (ApoE), which works as an acceptor for cellular cholesterol, impairs cholesterol efflux, resulting in the enrichment of cholesterol in lipid rafts [70]. This translates in an increased distribution of GM-CSF receptors in these regions, leading to increased response toward GM-CSF and therefore increased myelopoiesis [25,70]. A similar phenotype is observed, also when cholesterol transporters involved in cholesterol efflux are absent leading to intracellular accumulation of free cholesterol; this is the case of ApoE, ABCA1 and ABCG1, which are sensitive to LXR, and whose deficiency enhances the sensitivity of key receptors for myeloid promoting growth factor (that is the IL3/GM-CSF signaling), it promotes the commitment (via phosphorylation of Extracellular Signal-Regulated Kinases (ERK1/2) and Signal Transducers and Activator of transcription 5 (STAT5) and it increases circulating inflammatory cells and atherosclerosis [70,71]. Of note membrane receptors hyper-responsiveness is also conserved and shared by other myeloid-derived cells, such as the case of dendritic cells from apoE deficient mice which present increased MHC-II membrane clustering and activity [72].

While reduced lipoprotein uptake related to LDL-R deficiency is associated with a lower proportion of hematopoietic precursors resident in the bone marrow together with a more pronounced shortening of telomere length (TL), cholesterol accumulation impacts HSCs commitment. Indeed, the deficiency of Lysosomal Acid Lipase (LAL), the key enzyme which processes lipoproteins to cleave esterified cholesterol but also TGs to generate free cholesterol and FAs [73], results in cholesterol accumulation in the lysosome and abnormal number of HSCs, CMPS and GMPs, with enhanced capacity to form colonies within the niches and displaying reduced expression of apoptotic and checkpoints proteins [74]. In experimental models, LAL deficiency results into elevated circulating myeloid subsets, with elevated infiltrating capacity into inflamed tissues and improved ability to suppress lymphoid T cells proliferation [74]. These observations have been paralleled by the association of a number of cases of complete LAL deficiency (Wolman disease) to secondary hemophagocytic lymphohistiocytosis diagnosis, a pathological condition characterized by splenomegaly, elevated inflammatory markers (especially ferritin), in the absence of infection, and characteristic accumulation of foamy macrophages [75–78].

The inhibition of intracellular cholesterol synthesis by statins affects the epigenetic reprogramming and the hyperactivation of myeloid progenitors probably by interfering with lipid rafts abundance. Despite this observation, in the clinical setting, when patients were treated for three months with statins, no differences in precursors activation and trained immunity phenotype were observed [79]. This finding questions the possibility of repurposing metabolic drugs to target HSCs as their pharmacokinetics will largely influence the ability to reach specific tissues and cells. In vivo, the lack of statins efficacy could be explained either by the possibility that the inhibition of cholesterol biosynthesis in HSCs is counterbalanced by increased LDL-R expression and lipoprotein uptake, and/or by the fact that acute drop in cholesterol plasma levels would overcome the “priming effect” induce by long-term hypercholesterolemia.

Beyond their role as fuel for FAO, fatty acid metabolism shapes hematopoietic cells function as well. In fact, deficiency of Lipoprotein Lipase (LPL), that catalyses the hydrolysis of the triacylglycerol component of circulating chylomicrons and very low-density lipoproteins thereby providing free fatty acids to the cells, results in reduced expression of GM-CSF from macrophages and reduced myelopoiesis [80,81]. Likewise, the deficiency of Angiopoietin-like 4 (Angptl4), a constitutional LPL inhibitor, in HSCs promotes the expansion of the myeloid compartment under inflammatory conditions, paralleled by increased CD36 expression and reduced ABCG1 expression on macrophages [82,83]. This effect extends the formation of inflamed atherosclerotic lesions in mice fed on high fat diet [83]. Similarly, a microRNA-based approach, knocking-down (PPAR)-δ, reduces the transcription of Angptl4 and attenuates systemic inflammation, reduced the expression of CCR2 in monocytes, the expression of Monocyte Chemoattractant Protein-1 (MCP-1) and IL-1β in atheroma-resident macrophages and promoted the regression of atherosclerotic lesions in mice [84].

In addition, FAs act as building blocks for several macromolecules, including sphingolipids and phospholipids, which participate in HSC engagement and mobilization during atherosclerosis (Figure 3). Sphingolipids, for example, promote the activity of several transcription factors (PU.1, GATA1 and GATA2), leading to the reprogram of erythroid-primed MPPs towards the myeloid lineage under inflammatory conditions [85]. This effect is abrogated by Sphingosine-1-Phosphate (S1P); S1P itself demonstrated important role in the mobilization of HSC and their homing in peripheral sites (including spleen) [86,87]. Also, mice with elevated circulating S1P levels due to the genetic deletion of sphingosine kinase 2 (SphK2) [88] on an LDLR Knock-Out (KO) atherogenic background, showed reduced vascular endothelial monolayer permeability to LDL and monocytes recruitment within the plaque [89]. Moreover, phospholipids promote HSCs self-renewal and retention in the niche through cyclooxygenase (COX) dependent Prostaglandin E2 (PGE2) [90]) or lipoxygenases (LOX) dependent hydroxyeicosatetraenoic acid (HETE) production [91].

All together these evidences demonstrate the intimate connection between intracellular lipid metabolic reprogramming and hematopoietic cell fitness. Conditions that affect lipid homeostasis both at the systemic and cellular level might impact the proliferative and differentiating potential of hematopoietic cells, thus leading to changes in the flux from the bone marrow to the circulation of hyper-responsive committed cells that in turn might influence the progression of inflammatory-based disease, as in the case of atherosclerosis.

## Conclusions

Atherosclerosis results from unbalanced lipid metabolism coupled to inflammation. While lipid lowering therapy represents the pillar for decreasing circulating LDL-C levels, novel strategy should aim to improve the immunoinflammatory response. A general anti-inflammatory approach has been shown to improve cardiovascular outcome but also to affect general immune response toward infections. This implies that more tailored approaches are needed to target immune cells in the context of atherosclerosis. While lipid-lowering agents have demonstrated to possess several immunometabolic function in vitro, whether these drugs could reach a reasonable concentration to target immune cell without systemic side effects in vivo is debated.

In parallel, the identification of metabolic “checkpoints” that couple the reprogram of energetic machinery with immune cell functionality may offer innovative ways to target the inflammatory response associated to atherosclerosis. This is the case of (i) ACLY inhibition that by, reducing the acetyl-CoA pool required for histone acetylation, affects macrophage epigenetic program thus regulating TLR-driven gene expression after LPS stimulation [92,93]; (ii) LAL activity induction to boost the anti-inflammatory potential of macrophages [94]; (iii) PPAR-δ antagonism that reduces macrophage IL-1β expression [95]. At the same time, the novel understanding of immune-metabolic crosstalk in HSCs could contribute to depict collateral effects of developing lipid-lowering therapies, as the recent application of Angptl4 inhibitors for the treatment of hypertriglyceridemia that, despite promising, could bear activation of LPL in macrophages thus promoting their inflammatory activation [84].

The growing interest in HSCs biology has proposed that most of the above described immunometabolic events could be the result of metabolic and functional plasticity of precursor cells in the bone marrow. However, as the process of trained immunity can teach, targeting HSC commitment could be a double-edged sword. In fact, while priming of immune cell progenitors could be beneficial in diseases, where a boost of immune response is required to counteract a tolerogenic response (e.g. in cancer), it could vice versa be detrimental in cardiovascular diseases, where excessive inflammation contributes to disease progression. In this view, the expanding knowledge of metabolic and molecular circuits adopted by HSCs would offer innovative pharmacological target to control the activation of mature immune cells in the context of cardiovascular diseases.

## Abbreviations

HSCs: hematopoietic stem cells
MPPs: multi-potent progenitors
ApoE: apolipoprotein E
LDLR: low density lipoprotein receptor
BMAT: bone marrow adipose tissue
TCA: tricarboxylic acid cycle
OXPHOS: oxidative phosphorylation
FAO: fatty acid oxidation
PPAR-δ: Peroxisome proliferator-activated receptor delta
LAL: lysosomal acid lipase
CMPs: common myeloid progenitors
GMPs: granulocyte/macrophage progenitors
LXR: liver-X receptor
ABCA1: ATP binding cassette A1
ABCG1: ATP binding cassette G1
ApoC: apolipoprotein C
BMAT: Bone Marrow Adipose Tissue
LPL: lipoprotein lipase
GM-CSF: granulocyte-macrophage colony-stimulating factor
SDF1: Stromal Derived Factor-1
Angptl4: angiopoietin-like 4
LPL: Lipoprotein Lipase
CCR2: C-C chemokine receptor type 2
MCP-1: monocyte chemoattractant protein-1
NLRP3: NOD-Like Receptor Protein 3
S1P: Sphingosine-1-Phosphate
IL: Interleukin
TLR: Toll-Like Receptor
LPS: Lipopolysaccharide
